# Downregulation of Akt induces proximal tubule epithelial cell apoptosis via Foxo-1-BIM pathway in proteinuric states

**DOI:** 10.21203/rs.3.rs-6234375/v1

**Published:** 2025-04-25

**Authors:** Richard Chhaing, Qing Ma, Meredith Schuh, Elif Erkan

**Affiliations:** 1 Cincinnati Children’s Hospital

## Abstract

Proteinuria is a widely utilized surrogate marker in clinical practice for its predictive and prognostic value. The mechanistic link between proteinuria and progression remains elusive. Proximal tubule epithelial cells(PTEC) retrieve albumin in the glomerular filtrate via receptor mediated endocytosis facilitated by megalin-cubilin complex. We reported that cell-survival protein, Akt phosphorylates cargo binding endocytic adaptor protein to megalin, disabled-2(Dab2). We hypothesize that downregulation of Akt signaling as a result of overwhelmed endocytic machinery in albumin overload is linked to PTEC apoptosis in proteinuric states.

We show that cell culture and animal model of albumin overload inhibited phosphorylation of Akt in association with apoptosis in PTEC. Chemical inhibition and overexpression of Akt by constitutively active Akt plasmid exacerbated and alleviated apoptosis respectively in response to albumin overload in PTEC. Mouse with targeted inhibition of Akt1 and Akt2 in PTEC (Akt1/2lox/loxSGLT2cre) displayed perturbed albumin endocytosis at baseline. Albumin overload in Akt1/2 ^lox/lox^ SGLT2cre mouse led to dephosphorylation and translocation downstream Akt target, Forkhead box O-1 (Foxo1) to nuclei driving transcriptional activation of proapoptotic BIM followed by translocation of proapoptotic Bax and BIM to mitochondria and cytochrome-c to cytosol. In an effort to investigate the role of Akt in progression, we examined kidney biopsy specimens of patients with focal segmental glomerulosclerosis (FSGS) and minimal change disease. Kidney biopsies of patients with FSGS exhibited decreased pSer473-Akt expression in PTEC early in the course of disease, preceding progression to end stage kidney disease.

We conclude that downstream dephosphorylation of Foxo and transcriptional activation of BIM and subsequent mitochondrial injury drives apoptosis following Akt downregulation in PTEC albeit inhibition of albumin endocytosis in proteinuric states.

## Translational statement

There is a well-recognized relationship between the degree of proteinuria and progression in glomerular diseases, but the mechanism is poorly understood. In this study, we show that expression of survival protein Akt is downregulated in PTEC in response to albumin overload and in human glomerular disease. We demonstrate that inhibition of Akt promotes dephosphorylation of its downstream target Foxo1 and nuclear translocation of propaptotic BIM, leading to intrinsic apoptosis. We propose that interventions to restore Akt signaling will impede tubular injury by inhibiting PTEC apoptosis in proteinuric states.

## INTRODUCTION

Proteinuria is a widely accepted surrogate marker for monitoring disease activity and predicting progression in glomerular diseases particularly because of its association with tubulointerstitial fibrosis and tubular atrophy. Clinical studies have clearly demonstrated a direct relationship between progression and degree of proteinuria in glomerular kidney disease (1-3). Nephrotic milieu particularly albumin in the glomerular filtrate has long been identified as a potential mediator of tubular injury and inflammation. Since albumin accounts for the majority of the urinary protein that passes across the defective glomerular filtration barrier, research efforts have been focused on deciphering the mechanism of albumin overload induced PTEC injury in glomerular diseases.

Albumin in the glomerular filtrate is retrieved by PTEC via receptor mediated endocytosis. Despite possessing a high-capacity megalin-cubilin receptor complex, albumin concentration in the glomerular filtrate often reaches beyond the endocytic capacity of PTEC in glomerular diseases. This supraphysiologic concentration of albumin triggers proinflammatory, profibrogenic and apoptotic response in PTEC (4-7). In *in-vitro* experiments, high concentrations of albumin mimicking the nephrotic milieu induces upregulation of proinflammatory and profibrogenic genes and cytogenic RANTES by NF-kappa β in human kidney PTEC causing tubulointerstitial inflammation and fibrosis (5, 8). In an *in-vivo* albumin overload model generated by intraperitoneal albumin injections in mouse with or without unilateral nephrectomy, profibrogenic, monocyte chemoattractant factor, osteopontin, TGF β1 and tissue inhibitor of metalloproteinases-1 genes were upregulated (9, 10). Albumin overload *in vitro* triggers PTEC apoptosis (7, 11). In summary, PTEC apoptosis, being a precursor to tubular atrophy and chronic tubulointerstitial changes (has been at the epicenter of progressive kidney disease 12-14). Tubular apoptosis correlates with the degree of proteinuria predicting progression in patients with FSGS (15). Albumin overload induces mitochondrial injury by altering mitochondrial membrane potential and translocation of cytochrome-c from mitochondria to cytosol and Bax from cytosol to mitochondria causing apoptosis in PTEC (16). However, cell signaling pathways linking PTEC apoptosis to albumin endocytosis remain unexplored.

Akt is one of the pivotal molecules involved in cell signaling events regulating the cross talk between cell survival, proliferation and apoptosis (17). Akt is a serine and threonine protein kinase that inflicts its cell signaling effects by phosphorylating proteins following their translocation to the plasma membrane. Inactive cytosolic Akt is recruited to the plasma membrane engaging with PIP3 at the plasma membrane via PH domain. This leads to phosphorylation of Thr308 and Ser473 and activation of Akt by PDK1 and mTORC2 (18, 19). Forkhead box O transcriptional factors, Foxo1 and Foxo3 are downstream targets of Akt. Akt inhibits nuclear translocation, thus transcriptional activity of Foxo proteins by phosphorylation. Downregulation of Akt induces activation and translocation of Foxo proteins from cytoplasm to nucleus stimulating transcriptional activation of mitochondrial apoptotic proteins BIM and Bax (20). Albumin overload, mimicking proteinuric states results in downregulation of Akt expression in PTEC (21, 22).

In this study, our goal is to elucidate the upstream signaling pathways that lead to PTEC apoptosis in cell culture and animal model of albumin overload. We investigated the role of Akt and its downstream regulatory targets in albumin induced apoptosis in PTEC. We hypothesized that Akt signaling plays an important role in regulating the balance between survival and apoptosis in PTEC in response to albumin overload and this response is intertwined with its role in albumin endocytosis. We tested our hypothesis in *in-vivo* and *in-vitro* models of albumin overload and on patient kidney biopsies with FSGS.

## METHODS:

### Cell culture and in-vitro albumin overload:

Human kidney proximal tubule clone-8 (HKC-8) cells (courtesy of Dr. Racussen, John’s Hopkins University) were grown in Dulbecco’s modified Eagles serum/F12 (Life Technologies) supplemented with 5% certified fetal bovine serum, 100 U/ml penicillin, and 100 U/ml streptomycin (23).

In-vitro albumin overload model was included in Supplemental data. Preparation of cell lysates was explained in detail in supplement (21).

#### Antibodies:

The list of antibodies used for the experiments are provided as supplemental data.

### Western blotting:

Nuclear and cytoplasmic extraction was performed by NE-PER kit (Thermofisher). Mouse kidney cortex lysates were probed with cleaved caspase-9 and procaspase 3 antibodies (Cell Signaling). Mouse kidney mitochondria and cytosol were isolated by mitochondria isolation kit (Thermofisher) and expression of BIM, Bax and cytochrome-c was investigated by western blotting. Uncropped images were provided under supplemental data.

### Apoptosis assays:

HKC-8 cells were incubated with serum free media for 16 hours prior to albumin overload as indicated above. Cells were pretreated with pan Akt inhibitor MK-2206 (1uM) during albumin overload. HKC-8 cells were transfected with plasmids to overexpress constitutively active Akt, mutant Foxo1 or mutant Foxo3, 24 hours prior to albumin overload. The degree of apoptosis was evaluated by caspase-3 assay fluorometric assay (BD Biosciences). Protein expression was examined by western blotting using total Akt, Akt-pSer 473, Akt-pTh308, BIM, Bax and cytochrome-c (Cell Signaling) antibodies.

### Plasmids:

HKC-8 cells were transfected by CMV-Constitutively active (CA) PKB/Akt (courtesy of David Cook, University of Melbourne), Foxo1 (pcDNA3 Flag FKHR AAA mutant was a gift from Kunliang Guan, Addgene plasmid # 13508) and Foxo3 plasmids with mutations at the Akt phosphorylation sites (FLAG-FOXO3 6A was a gift from Anne Brunet, Addgene plasmid # 24382) plasmid using Lipofectamine (24-26). HKC-8 cells were transfected with a plasmid encoding GFP tagged Foxo1 (GFP-Foxo1 was a gift from Domenico Accili, Addgene plasmid # 17551) prior to albumin overload experiments to visualize translocation of Foxo1 to nucleus(27). Nuclei were costained by Hoechst 3342 counterstaining. Nuclear translocation of GFP-Foxo1 was evaluated by calculating corrected nuclear fluorescence in HKC-8 cells treated with albumin and controls by using ImageJ.

Chromatin immunoprecipitation (CHIP) experiments: Ez-CHIP kit (Millipore) was used to investigate Foxo1-BIM DNA interaction. Details of the CHIP experiments can be found in Supplementary data.

Explanation of the animal model of albumin overload was included in Supplemental data.

### Generation of mouse with tissue specific deletion of Akt1 and Akt2 in PTEC:

Akt1/2 ^lox/lox^ SGLT2 cre(+) mouse was generated by crossing Akt1^lox/lox^ and Akt2 ^lox/lox^ mouse (courtesy of Morris Birnbaum) with SGLT2 cre positive mouse (courtesy of Manoocher Soleimani) (28). Targeted deletion of Akt1 and Akt2 in PTEC was performed on SGLT2 cre mouse because it targets S1 segment of PTEC which is the main segment where albumin endocytosis takes place. Albumin overload was introduced by intraperitoneal albumin injections as outlined above in Akt1l^ox/lox^ Akt2 ^lox/lox^ SGLT2 cre(+) mice and cre(−) littermates.

Animal protocols were approved by Cincinnati Children’s Hospital IACUC. Anesthesia was introduced by 5% isoflurane. Euthanasia was performed by major organ harvest and cervical dislocation. All experiments were performed in accordance with CCHMC IACUC guidelines and regulations. The authors complied with the ARRIVE guidelines.

Details of immunofluorescence staining was included in Supplemental data.

Patient biopsy samples were obtained from CCHMC DISCOVER TOGETHER databank. All methods were carried out in accordance with relevant guidelines and regulations. All experimental protocols were approved by CCHMC IRB. Informed consent was obtained from all subjects and/or their legal guardian(s).

Patients had estimated GFR >60ml/min/1.73m^2^ (by modified Schwartz formula) at the time of the diagnostic kidney biopsy. Exclusion criteria: Secondary FSGS, and patients with low GFR to eliminate potential confounding effect of chronic kidney disease on pathology. Urine protein:creatinine (mg:mg) (Upc) ratio was measured on spot urine collections at the time of clinic visits.

Detailed method for quantification of Akt p-Ser473 expression on patient kidney biopsies was included in Supplemental data.

### Statistical analysis:

SPSS program was used for statistical interpretation of the data. Results of three or more experiments were expressed as mean±SEM.

The statistical difference between groups was assessed by unpaired samples t-test. Statistics for not normally distributed data were performed by Mann Whitney U test for comparison between two groups. Normality of the data was determined by SPSS Using Kolmogorov-Smirnov test. One way ANOVA was used to compare multiple groups for normally distributed data whereas Kruskal-Wallis test was used for data that were not normally distributed.

The density of Western blot bands was evaluated by Image J software. P <0.05 was considered statistically significant.

Data sharing questionnaire is included in the submission. Data that support the findings are provided within the manuscript and supplementary information files.

## RESULTS:

### Akt regulates proximal tubule epithelial cell apoptosis in albumin overload:

**Figure-1:**
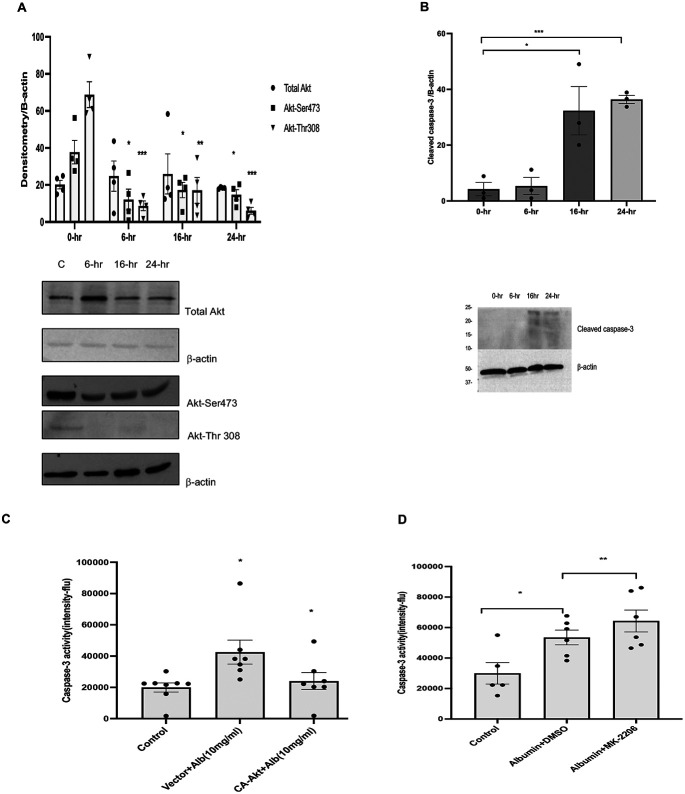
Serine-threonine kinase Akt regulates proximal tubule epithelial cell apoptosis in albumin overload: Fig-1a-Immortalized human proximal tubule epithelial cells (HKC-8) are treated with endotoxin free human albumin (10mg/ml) for 6, 16 and 24 hours. Expression of total Akt, Akt-Ser473 and Akt-Thr308 is examined by Western blotting. Ser473Akt and Akt-Thr308 expression is downregulated as early as 6 hours followed by 16 and 24 hours (n=4). Fig-1b-Apoptotic activity measured by cleaved caspase-3 expression started at 16 hours of albumin overload maximized at 24 hours (n=3). Fig-1c, d-Overexpression of Akt by constitutively active Akt plasmid decreased apoptosis, in comparison to HKC-8 cells transfected by the vector (n=5) whereas inhibition of Akt expression by pan Akt inhibitor MK-2206 (1uM) decreased apoptosis (Fig-1d) (n=5), measured by fluorometric caspase-3 assay. Independent samples T-test was used for comparison. * = p<0.05, ** = p<0.01, ***= p<0.001

We determined total and phosphorylated Akt expression and cleaved caspase-3 activity in HKC-8 cells treated for 6, 16 and 24 hours with albumin (10mg/ml) and compared them with baseline (0-hr) levels. There was a significant increase in PTEC apoptosis in correlation with exposure between groups (p<0.05). We demonstrated that albumin overload caused downregulation of Akt-serine 473 and Akt-threonine 308, in association with an increase in apoptosis tested by cleaved caspase-3 activity in HKC-8 cells (Fig-1a, b). Interestingly, decrease in phosphorylation of Akt preceded apoptosis suggesting a causal relation. The apoptotic response increased with the duration of albumin overload and peaked at 24 hours. Next, we investigated the causality between Akt expression and PTEC apoptosis by measuring caspase 3 activity in conjunction with inhibition or activation of Akt in HKC-8 cells subjected to albumin overload for 24 hours. We inhibited and overexpressed Akt with a pan Akt inhibitor MK2206 and CA-Akt plasmid. Overexpression of Akt by CA-Akt plasmid caused a decrease in albumin induced apoptosis whereas inhibition of Akt activity revealed an increase in caspase-3 activity measured by fluorometry in response to albumin overload indicating the pivotal role of Akt in maintaining cell survival counterbalancing apoptosis (Fig-1c, d).

### Albumin overload causes a decrease in pSer473Akt expression in association with PTEC apoptosis *in-vivo*:

**Figure-2:**
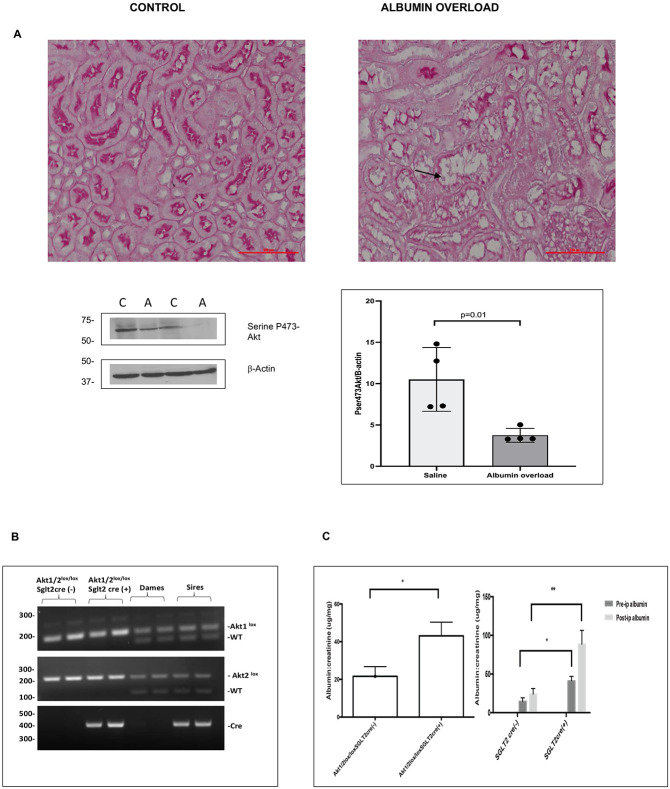
Albumin overload induces proximal tubule epithelial cell apoptosis in association with inhibition of phosphorylated Akt expression in-vivo: Fig-2a-Wild type C57BL/6 mice and littermate controls (6-8 weeks old) underwent intraperitoneal albumin (low-endotoxin BSA, 10 mg/g body weight, dissolved in saline) for 5 consecutive days per week or normal saline injections for 6 weeks (n=4). Albumin overload caused proximal tubule cell apoptosis evidenced by sloughed off proximal tubule epithelial cells with condensed nuclei in the tubular lumen with H&E staining on light microscopy in association with decreased Akt pSer437 expression Fig-2b-Targeted deletion of Akt1 and Akt2 in PTEC was accomplished by generating a double knock out mice, Akt1/2 ^lox/lox^ SGLT2 cre+. Fig-2c-Akt1/2 ^lox/lox^ SGLT2 cre+ mouse displayed mild increase in urine albumin levels at baseline. Akt1/2 ^lox/lox^ SGLT2 cre+ mice displayed a 2.15-fold increase in urinary albumin excretion in comparison to 1.6-fold increase in cre-animals in response to albumin overload. Independent samples t-test was used for comparison * p<0.05 ** p<0.01. C-Control A-mice received intraperitoneal albumin

C57BL/6 mice subjected to albumin overload by intraperitoneal albumin injections displayed PTEC apoptosis. H&E staining of mouse kidney tissue exhibited sloughed off brush borders and rounded up PTEC with condensed nuclei pathognomonic of tubular injury and apoptosis. Western blot analysis of kidney cortex lysates showed a decrease in pSer473 Akt expression in mice subjected to albumin overload (Fig-2a).

### Targeted deletion of Akt1 and Akt2 in proximal tubule epithelial cells exacerbates PTEC apoptosis via mitochondrial pathway in response to albumin overload:

We explored the role of Akt in cell survival in a mouse model with targeted deletion of Akt1 and Akt2 in PTEC. Akt has three isoforms, Akt1 is mainly expressed in PTECs and Akt2 is expressed in PTEC and predominantly in podocytes (29-31). Expression of Akt 3 is restricted to testes and brain. We inhibited both Akt1 and Akt2 expression in PTECs because of the high level of redundancy between these two isoforms. We generated a mouse with targeted deletion of both Akt1 and Akt2 (Akt1^lox/lox^/Akt2^lox/lox^) in PTEC by utilizing SGLT2 promoter (Fig-2b). Akt1^lox/lox^/Akt2^lox/lox^ mouse displayed normal histology with inhibition of Akt1 and Akt2 shown by immunohistochemistry (Suppl-Fig-2a,b). Akt1^lox/lox^/Akt2^lox/lox^ SGLT2cre(+) mouse exhibited baseline albuminuria confirming the role of Akt in albumin endocytosis and this was exacerbated by intraperitoneal albumin injections (Fig-2c). Akt1^lox/lox^/Akt2^lox/lox^ SGLT2cre(+) mice displayed 2.15-fold increase in albuminuria as a response to albumin overload in comparison to 1.6-fold in controls. Akt1^lox/lox^/Akt2^lox/lox^ SGLT2cre(+) maintained normal kidney function (Suppl-Fig-2c)

**Figure-3:**
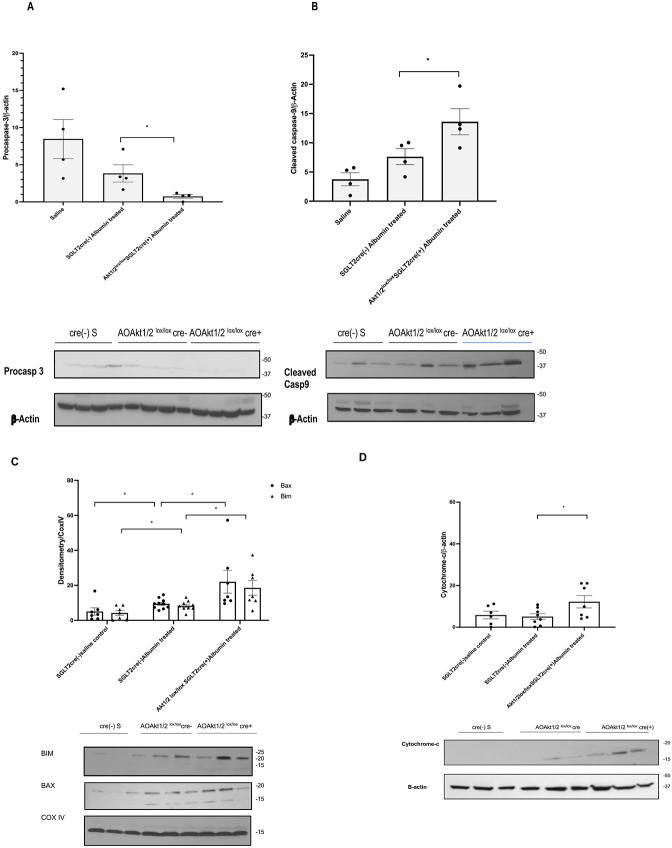
Targeted deletion of Akt1 and Akt2 in proximal tubule epithelial cells induces intrinsic apoptotic pathway with albumin overload: Fig-3a, b-Western blotting of kidney cortex lysates of Akt1/2^lox/lox^ SGLT2 cre+ mice exhibited decreased procaspase 3 and increased cleaved active caspase-9 activity in response to albumin overload (n=4). Independent samples t-test was used for comparison. Fig-3c, d-Mitochondrial and cytosolic isolates from kidney cortex of albumin overloaded Akt1/2^lox/lox^ SGLT2 cre+ mice exhibited mitochondrial translocation of Bax and BIM and cytosolic translocation of cytochrome-c consistent with increased apoptosis via mitochondrial pathway. Student-t test was used to compare cytosolic cytochrome-c expression between controls (n=6), Akt1/2^lox/lox^ SGLT2 cre-(n=8) and Akt1/2^lox/lox^ SGLT2 cre+(n=7). Mann-Whitney U test was used for statistical comparison of two independent samples to compare mitochondrial expression of Bax and BIM (n=7). Kruskal-Wallis and ANOVA test showed difference among groups (p<0.05). S=saline injected control AO: Albumin overload * p<0.05

We examined the role Akt in PTEC apoptosis by inducing albumin overload in Akt1^lox/lox^/Akt2^lox/lox^ SGLT2cre(+) and cre(−) littermates. Apoptosis was evaluated by procaspase-3 and cleaved caspase-9 activity in kidney cortex lysates. Inhibition of Akt1 and Akt2 in PTEC resulted in a significant decrease in procaspase-3 and increased cleaved caspase-9 activity (Fig-3a, b). These findings, in line with cell culture experiments, confirmed the role of impaired Akt activity tilting the balance to PTEC apoptosis in response to albumin overload.

In order to further delineate the mechanism of albumin overload induced apoptosis in relation to Akt downregulation in PTEC, we examined Bax, BIM and cytochrome-c expression in mitochondrial and cytosolic isolates from renal cortex of Akt1^lox/lox^/Akt2^lox/lox^ cre(+) mice and control littermates. Western blotting of the mitochondrial isolates and cytosol revealed translocation of proapoptotic Bax and BIM to the mitochondria and cytochrome-c to the cytosol in response to albumin overload in Akt1^lox/lox^/Akt2^lox/lox^SGLT2 cre(+) mice confirming activation of mitochondrial intrinsic apoptotic pathway (Fig-3c, d). We concluded that inhibition of Akt activity in PTEC potentiates apoptotic effect of albumin overload in PTEC by causing mitochondrial alterations.

### Albumin overload in PTEC decreases phosphorylation of downstream Akt targets Foxo-1 and Foxo-3:

**Figure-4:**
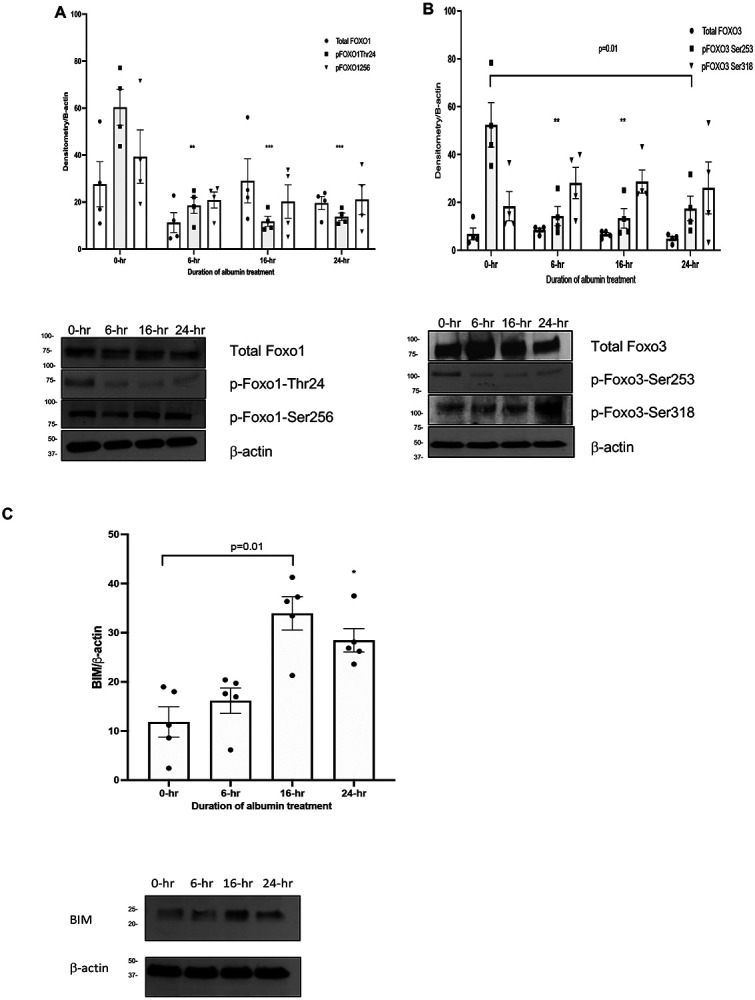
Albumin overload in PTECs cause downregulation of Akt activity and phosphorylation of its downstream targets Foxo1 and Foxo3: Fig-4a-c-HKC-8 cells are incubated with endotoxin free human albumin (10mg/ml) for 6, 16 and 24 hours and probed to investigate phosphorylation of Foxo1 and Foxo3. Independent samples t-test was used for statistical comparison between two groups. Phosphorylation of Foxo1 at Ser 24 and Foxo3 at Ser 253, both representing Akt phosphorylation sites were diminished starting at 6 hours in association with proapoptotic BCL-2 family protein, BIM expression by Mann-Whitney U test. (n=5) * = p<0.05, ** p<0.01, *** p<0.001

Akt and Akt related serine, threonine kinases regulate cell survival by inactivating transcriptional proteins via phosphorylation. Phosphorylation of Foxo1 and Foxo3 by Akt inhibits their translocation to the nucleus thus transcription of proapoptotic BIM promoting cell survival and proliferation (32). *We hypothesize that inhibition of Akt activity will hinder phosphorylation of Foxo proteins allowing uninhibited transcriptional activation of proapoptotic protein BIM in response to albumin overload*. In order to test this hypothesis, we explored the role of decreased phosphorylation of Foxo1 and Foxo3 by Akt in albumin induced apoptosis in PTEC. As a first step, we examined total Foxo1 and Foxo3, p-Foxo1-Thr24, p-Foxo1-Ser 256, p-Foxo3-Ser-253 and p-Foxo3-Ser-318 expression in response to albumin overload in PTEC at 6, 16 and 24 hours. Phosphorylation of Foxo1 and Foxo3 at Akt phosphorylation sites, Thr-24 and Ser-253 were diminished respectively as early as in 6 hours in response to albumin overload in PTEC (Fig-4a, b). Proapoptotic BIM activity increased at 6, 16 and 24 hours of albumin overload in concert with apoptosis (Fig-4c). The association between decreased Akt phosphorylation of Foxo1 and Foxo3 and increased BIM activity prompted us to investigate the potential causality between BIM transcription and increased nuclear translocation of Foxo proteins.

### Albumin overload induces nuclear translocation of Foxo-1 and transcription of BIM by Foxo-1:

**Figure-5:**
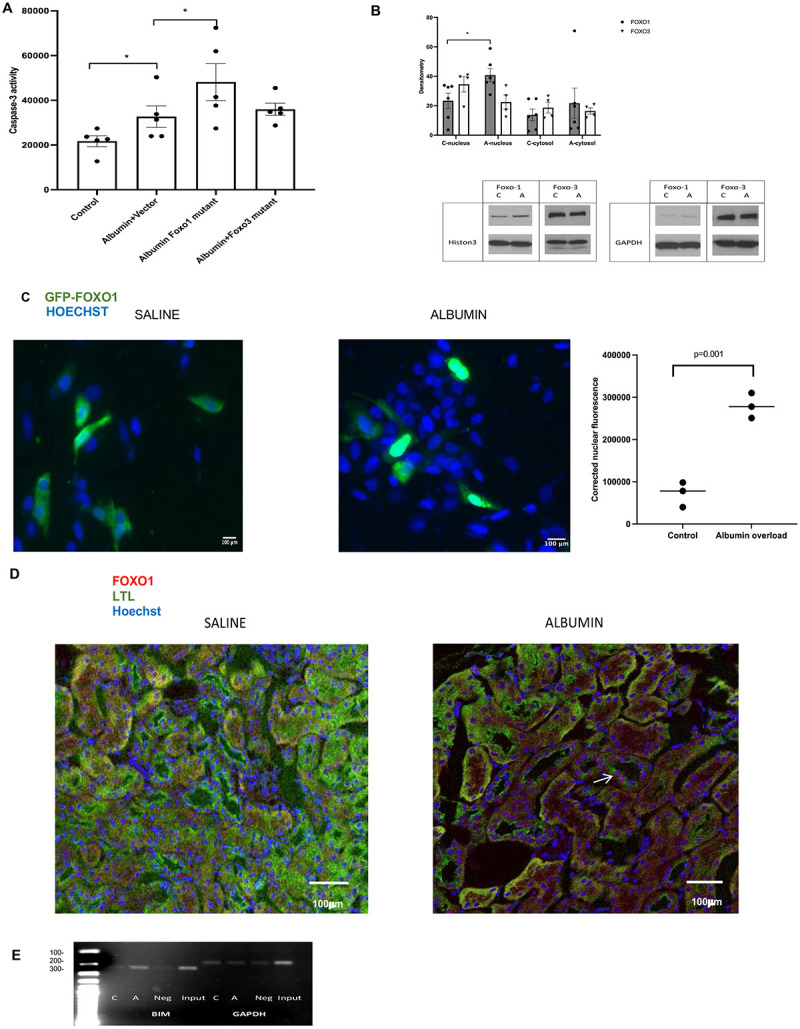
Inhibition of phosphorylation by Akt and nuclear translocation of Foxo1 promotes apoptosis in association with increased BIM transcription in PTECs in response to albumin overload: Fig-5a-HKC-8 cell transfected with plasmids possessing mutations at Akt phosphorylation sites of Foxo1 and Foxo3 were subjected to albumin overload for 24 hours (n=5) Inhibition of Foxo1phosphorylation by Akt augmented PTEC apoptosis induced by albumin overload. Independent samples T test was used for comparison of two groups. * p<0.05, ** p<0.01 Fig-5b-Nuclear and cytosolic Foxo1 and Foxo3 expression in response to albumin overload was examined by western blotting. Cytosol and nuclei were isolated after 24 hours of albumin overload in PTECs. Foxo1 but not Foxo3 translocated to nuclei in association with albumin induced apoptosis (n=6). Fig-5c-Proximal tubule epithelial cells transfected with GFP-Foxo1 displayed nuclear translocation of Foxo1 and apoptosis evidenced by nuclear condensation and rounded up cells (n=3). Fig-5d-We demonstrated nuclear translocation of Foxo1 in Akt1/2^lox/lox^ SGLT2 cre mice kidneys subjected to albumin overload In CHIP experiments, we investigated DNA-protein interactions. Nuclear extracts of PTEC subjected to albumin overload were immunoprecipitated with Foxo1 antibody. Fig-5e-The immunoprecipitated were subjected to PCR using primers against BIM promoters(n=3). GAPDH was utilized as housekeeping gene. Independent samples T test was used for comparison of two groups.

We explored the role of decreased Akt phosphorylation of Foxo proteins in albumin overload induced PTEC apoptosis. We transfected HKC-8 cells with Foxo1 and Foxo3 plasmids possessing mutations at Akt phosphorylation sites to dismantle their ability to be phosphorylated by Akt. Transfection of HKC-8 cells with mutant Foxo1 but not with mutant Foxo3 led to an increase in albumin-induced apoptosis evidenced by increased caspase-3 activity showing the role of Akt phosphorylation of Foxo1 in safeguarding PTEC from albumin induced apoptosis (Fig-5a). Foxo proteins exhibit their apoptotic activity by inducing transcription of proapoptotic genes in the nucleus. We next examined nuclear translocation and cytosolic expression of Foxo1 and Foxo3 in response to albumin overload in PTEC. Nuclear translocation of Foxo1 but not Foxo3 was augmented in response to albumin overload indicating that Foxo1 is involved in transcriptional regulation of albumin-induced apoptosis (Fig-5b). In line with this finding, HKC-8 cells transfected with GFP-Foxo1 exhibited translocation of Foxo1 to the nucleus in response to albumin overload (Fig-5c). We further investigated nuclear translocation of Foxo1 in Akt1/2^lox/lox^ SGLT2cre(+) mice subjected to albumin overload. Akt1/2^lox/lox^ SGLT2cre(+) mice demonstrated nuclear translocation of Foxo1 in PTEC in response to albumin overload (Fig-5d).

We next investigated Foxo1 mediated transcription of BIM in response to albumin overload in HKC-8 cells. Chromatin immunoprecipitation experiments revealed increased Foxo1 induced transcription of BIM in association with albumin overload in PTEC (Fig-5e). The rest of the CHIP experiment images were provided as supplemental data (Suppl-Fig-5). We concluded that increased transcription of BIM by Foxo1 as a result of decreased phosphorylation by Akt induces apoptosis in response albumin overload in PTEC.

### Kidney biopsy sections of patients with FSGS display decreased expression of Akt-pSer473 in the proximal tubule epithelial cells in association with poor prognosis:

We next investigated the clinical relevance of our findings by examining Akt activation in a progressive human glomerular disease. FSGS is the most common and most challenging glomerular disease in children. It is characterized by nephrotic range proteinuria and rapid progression to end-stage kidney disease. We previously demonstrated that patients with FSGS exhibit apoptosis in PTEC in comparison to MCD preceding disease progression (15). We explored Akt expression in PTEC on patient kidney biopsy sections with primary FSGS, as a potential mechanism leading to apoptosis and thus progression. Patient kidney biopsies with MCD were used as control because it is a self-limited disease responsive to steroids.

**Figure-6:**
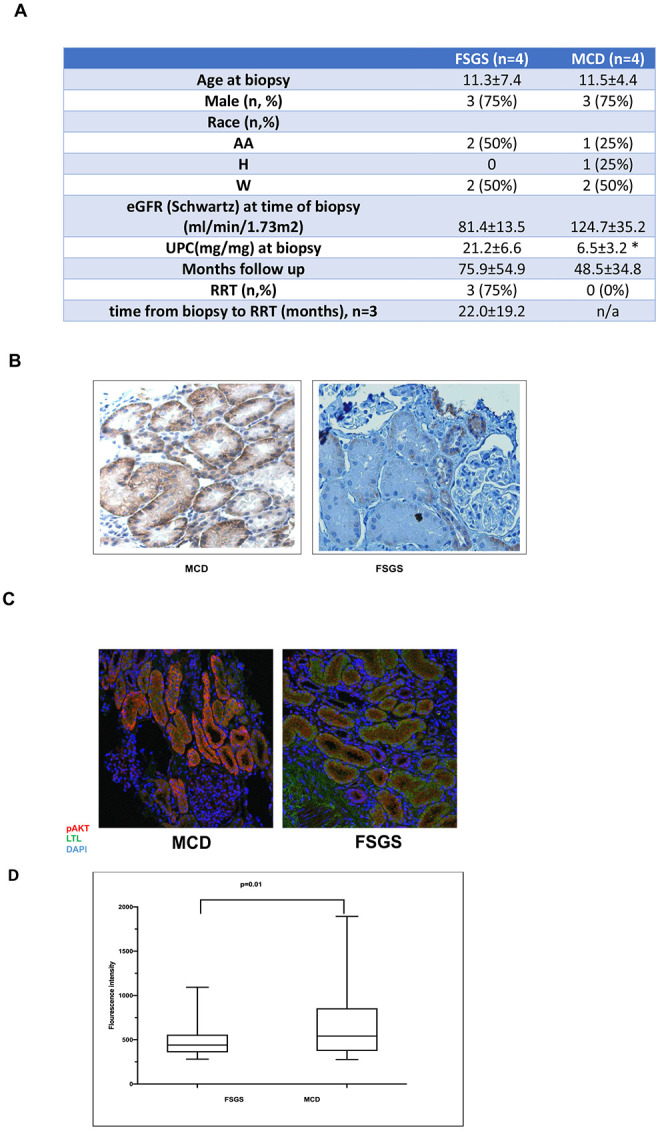
Akt expression is downregulated in patient kidney biopsies with focal segmental sclerosis (FSGS) preceding progression: Fig-6a-Pediatric patient kidney biopsies and demographic data of the patients with FSGS and minimal change disease (MCD) were examined. Demographics of the patient populations were comparable. FSGS patients had estimated GFR > 60ml/min/1.73m^2^ at the time of the kidney biopsy. Seventy five percent of the patients developed end stage kidney disease (ESKD) requiring renal replacement therapy (RRT) in FSGS group. Fig-6b-d-Quantitative evaluation of Pser473-Akt revealed decreased expression in PTEC in pediatric patient kidney biopsies with FSGS in comparison to MCD measured as signal intensity in PTEC under confocal microscopy (n=4) Mann-Whitney U test was used for statistical comparison of two groups. AA=African American H=Hispanic W=White UPC: Urine protein/creatinine ratio

Diagnostic patient kidney biopsies obtained at the time of initial presentation with an eGFR of >60ml/min/1.73m2 were enrolled. Patients had a minimum follow-up of five years. Demographic data were comparable (Fig-6a). We showed that PTEC displayed lower p-serine 473 Akt expression in patient kidney biopsies with FSGS in comparison to MCD by IHC (Fig-6b). We also investigated p-serine473 Akt expression by immunofluorescence staining in patient kidney biopsy samples with FSGS and MCD. Quantitative calculation of immunofluorescence intensity was performed by confocal microscopy as outlined in the methods section. Both patient groups demonstrated nephrotic range proteinuria (Upc>2) at the time of the kidney biopsy. Eighty percent of the patients with FSGS progressed to end-stage renal disease during further follow-up Patients with FSGS had significantly lower levels of pSer473-Akt expression in PTEC in comparison to patients with MCD preceding progression as measured based on fluorescence intensity (Figure-6c,d). Presence of decreased PTEC pSer473-Akt on diagnostic kidney biopsy sections at the time of eGFR of >60ml/min/1.73m2 prior to progression to ESKD indicates a causal link between diminished PTEC Akt activity and progression.

## Discussion:

Akt is one of the most versatile proteins involved in a wide variety of cell signaling events ranging from cell proliferation, migration, insulin signaling to survival. Dysregulation of Akt signaling is implicated in human diseases, most notably in cancer and diabetes mellitus. Akt has three different isoforms Akt1, Akt2 and Akt3 (33). Despite the great degree of homology (>80%) between Akt isoforms, knock-out (KO) mouse models demonstrated their diverse functions (34). Global KO of Akt1 in mouse causes defects in fetal and postnatal growth and increased mortality. Akt2 KO mouse displays a diabetes-like phenotype, whereas Akt3 KO has a negative impact on brain development. Previously we have reported that Akt regulates albumin endocytosis via phosphorylation of Dab2, adaptor protein to megalin (35). In this paper, we demonstrate a novel pathway that explains how Akt inhibition leads to PTEC apoptosis in cell culture and animal model of albumin overload. We further tested our hypothesis in a mouse model with targeted inhibition of both Akt1 and Akt2 as they are both expressed in PTEC. We utilized SGLT2cre for targeted inhibition of Akt1 and Akt 2 particularly in the S1 segment of PTEC where albumin endocytosis is mostly pronounced.

The proliferative and cell survival properties of Akt have been widely investigated in cancer research as most cancer cells exhibit unconstrained Akt activity and its downstream targets (36, 37). The main function of Akt in proliferation and cell survival is mediated via phosphorylation and inactivation of Foxo proteins (20). In cancer, dysregulated activation of Akt causes phosphorylation and inactivation of Foxo proteins resulting in their accumulation in the cytoplasm. Foxo proteins regulate cell cycle arrest, DNA repair and apoptosis. Akt phosphorylates Foxo1 at T24, S256, and S319, and Foxo-3a and Foxo-4 at three equivalent sites. Akt inhibits BH3 only proteins through its effect on transcriptional factors, Foxo1 and Foxo3. Phosphorylation of Foxo proteins by Akt translocate them to the cytoplasm and block their interaction with their proapoptotic target genes. Decreased phosphorylation and activation of Foxo proteins propagate cell cycle arrest and apoptosis by activation of Fas, Bad and BIM. Bax and Bak contain BH1-BH4 domains and mediate apoptosis by increasing permeability of mitochondrial outer membrane. BH3 only proteins Bid and BIM sense and convey pro-death signals by binding directly to Bax to induce its activation. By activation of BIM, it is constitutively inserted in the outer mitochondrial membrane via a C-terminal transmembrane anchor from where it can activate the effector of cytochrome c-release, Bax (38). Subsequent to activation of this molecular cascade, apoptosis ensues.

**Figure-7:**
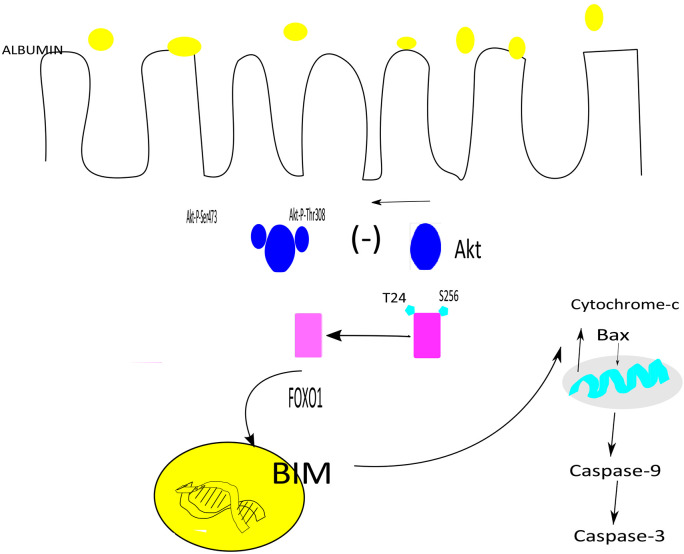


In this paper, we demonstrate that albumin overload mitigates phosphorylation and activation of Akt in *in-vivo* and *in-vitro* albumin overload experiments in association with apoptosis. To shed light to potential causality between Akt downregulation and apoptosis, we generated Akt1/2^lox/lox^ SGLT2cre(+). Targeted deletion of Akt1 and Akt2 in PTEC potentiated albumin overload induced apoptosis by perpetuating translocation of Bax and BIM to mitochondria and cytochrome-c to cytosol. We show that Akt downregulation resulted in dephosphorylation and activation of downstream target, Foxo1, promoting its translocation to the nucleus stimulating genetic expression of proapoptotic BIM, a major player in albumin induced mitochondrial apoptosis (Fig-7).

Underlying molecular mechanism leading to Akt downregulation in PTEC in response to proteinuria is unknown. *In vivo* studies by two photon microscopy demonstrated that sieving coefficient of PTEC for albumin is low at 0.003 and that PTECs directly retrieve few grams of albumin daily (39). There is body of evidence indicating that albumin endocytosis in PTEC is intertwined with cell signaling pathways. Albumin is internalized via megalin by receptor mediated endocytosis in PTECs. Biemesderfer et al have shown that megalin undergoes γ-secretase mediated regulated intramembrane proteolysis (RIP) culminating release of cytosolic domain of megalin, linking albumin endocytosis to gene regulation similar to Notch like signaling pathway (40, 41). In PTEC cell culture model, CRISPR/Cas9 knock-out of megalin (LRP-2) receptor resulted in increased transcription of proinflammatory genes suggesting a link between endocytic pathway and inflammation (42). Most recently, spatiotemporal analysis of the entire nephron by intravital imaging revealed induction of wide range changes in gene expression involved in endocytosis, cellular injury, cell growth and development in proximal tubule in response to proteinuria (43). Implications of Akt mediated cell signaling in the setting of chronic kidney disease (CKD) has been controversial and tissue specific. In CKD suppression of PI3K-Akt pathway resulted in muscle cell proteolysis which was restored by insulin administration (44). In early diabetic nephropathy and CKD loss of Akt activity in podocytes leads to apoptosis (45). On the contrary in the setting of diabetes mellitus, high glucose was shown to induce PI3-Akt and TGF-β-1 mediated activation of Akt contributing to epithelial mesenchymal transformation (46, 47). Our findings clearly demonstrate the role of cell signaling events orchestrated by Akt in PTEC apoptosis in response to albumin overload. Albumin overload results in inhibition of Akt expression in cell culture and animal models. We postulate Akt expression is inhibited as a negative feedback response originating from the overwhelmed endocytic network with albumin overload in PTEC. Mouse with targeted deletion of Akt1/2 in PTEC shows increased albuminuria which was exacerbated by albumin overload confirming that Akt signaling is involved in albumin endocytosis. Surprisingly, decreased albumin endocytosis induced by deletion of Akt1/2 in PTEC causes increase in apoptosis. We surmise that PTEC apoptosis is partly driven by the signaling pathways led by downregulation of Akt triggered by an overwhelmed endocytic machinery in addition to a direct toxic effect of internalized albumin in proteinuric states. Previous studies have demonstrated the role dysregulation of Mammalian Target of Rapamycin (mTOR) activity on PTEC with high albumin concentrations in association with decreased expression of megalin. Supraphysiologic albumin concentrations overactivated mTORC1 and inhibited mTORC2 activity through ERK/S6K/TSC2-dependent pathway whereas physiologic albumin concentrations activate Akt via mTORC2 (48). Furthermore, lithium treatment increased mTORC2 activity leading to the phosphorylation of Akt at Ser473 (49).

We previously reported that patients with FSGS exhibit increased PTEC apoptosis in comparison to MCD on their kidney biopsy samples (15). Patient kidney biopsies with FSGS exhibited decreased pSer473Akt expression in PTEC preceding disease progression. Patient kidney biopsies with FSGS rather than MCD exhibited downregulation of Akt expression in PTEC despite both patients displayed nephrotic range proteinuria. We surmise that this is because of short lived and treatment responsive nature of proteinuria in MCD. We tested our hypothesis on a small number of pediatric patient kidney biopsies, future studies are needed to further investigate the overlapping cell signaling events between albumin endocytosis and proteinuria induced apoptosis in a wider range of glomerular diseases.

In summary, our data support the role of decreased Akt expression in progressive proteinuric glomerular diseases. Upstream events that regulate Akt activity as part of the endocytic machinery requires further investigation. It is conceivable that maneuvers stimulating Akt activation in PTEC in proteinuric states can be beneficial to protect cells from apoptosis and further tubulointerstitial injury. However, institution of these agents requires very careful fine tuning to adjust the effective dose as overexpression of Akt can lead to uncontrollable cellular proliferation.

In conclusion, we described a novel molecular pathway that leads to PTEC apoptosis in proteinuric states. Future research will facilitate understanding the role of Akt signaling in PTEC apoptosis and tubulointerstitial injury thus progression of glomerular diseases and how this process is linked to albumin endocytosis.

## Data Availability

Data sharing questionnaire is included in the submission. Data that support the findings are included within the manuscript.
